# A cross-modal feature fusion model integrating tongue image phenotypes and accessible endocrine profiles for the screening of polycystic ovary syndrome

**DOI:** 10.3389/fmed.2026.1858582

**Published:** 2026-08-31

**Authors:** Chang Liu, Yuwei Luo, Tianzi Chen, Jie You, Jinping Wang

**Affiliations:** 1 Department of Hospitality and Business Management, Technological and Higher Education Institute of Hong Kong, Hong Kong, Hong Kong SAR, China; 2 School of Advanced Engineering, Great Bay University, Dongguan, China; 3 Basic Medical School of Naval Medical University, Naval Medical University, Shanghai, China; 4 Department of Information and Software Engineering, East China JiaoTong University, Nanchang, China; 5 Department of Traditional Chinese Medicine, Jiangsu Kunshan Hospital of Integrated Traditional Chinese and Western Medicine, Kunshan, China

**Keywords:** cross-modal feature fusion, multimodal clinical screening, polycystic ovary syndrome, risk stratification, tongue image phenotypes

## Abstract

**Background:**

Traditional PCOS assessment relies on the integration of clinical history, biochemical evaluation, and pelvic ultrasonography, which may limit the efficiency of preliminary screening in some clinical settings. This study aimed to develop an AI-assisted multimodal screening model by integrating tongue-image phenotypes with structured clinical, endocrine, and metabolic variables for PCOS risk assessment.

**Methods:**

We collected a multimodal dataset comprising standardized tongue images and corresponding demographic, anthropometric, reproductive, endocrine, and metabolic variables. First, a pre-trained deep learning model was utilized to extract objective tongue features, including color, texture, and coating characteristics. Subsequently, a cross-modal feature fusion framework was designed to integrate these digital tongue phenotypes with clinical data. Machine learning classifiers were employed to construct the binary classification model.

**Results:**

The experimental results demonstrated that the proposed cross-modal fusion model outperformed single-modal approaches (using either tongue images or clinical data alone). The model achieved an Area Under the Curve (AUC) of 89.20%, and a Sensitivity of 85.96%. Feature importance analysis revealed that specific tongue color parameters and menstrual cycle irregularities were the most significant predictors, consistent with clinical findings.

**Conclusion:**

The integration of tongue-image phenotypes with structured clinical, endocrine, and metabolic variables showed promising performance for PCOS screening in the internally validated cohort. The proposed model should be considered an adjunctive screening and risk-stratification tool rather than a replacement for routine clinical assessment and established diagnostic procedures.

## 1 Introduction

Gynecological diseases, particularly Polycystic Ovary Syndrome (PCOS), represent a substantial global health burden, affecting approximately 10–13% of women of reproductive age (1). The impact of PCOS extends far beyond reproductive dysfunction; it is a complex metabolic syndrome associated with a significantly increased risk of type 2 diabetes, cardiovascular diseases, and psychological distress (2). Given its progressive nature, early detection is of paramount importance for long-term chronic disease management and improving patients' lifelong health trajectories (3).

Currently, the gold standard for PCOS diagnosis relies on pelvic ultrasonography and serum hormone assays. However, these conventional methods face significant challenges in large-scale screening and primary care settings. First, ultrasound interpretation is inherently subjective, with inter-operator consistency often limited to a moderate level (*k* = 0.52 − 0.65). Second, factors such as obesity or acute stress can skew imaging clarity and biochemical markers, respectively. Most critically, resource disparities severely limit accessibility; data indicate that only 15% of primary care clinics in rural areas can perform these diagnostic workflows, leading to an average specialist waiting time of 4.2 ± 1.8 weeks (4). This gap in healthcare accessibility highlights the need for an efficient, objective, and clinically applicable adjunctive tool to support preliminary PCOS screening and risk stratification.

Computerized tongue-image analysis has emerged as a potentially useful imaging modality for providing complementary phenotypic information. However, tongue-image analysis alone is unlikely to provide sufficient information for PCOS diagnosis because of the heterogeneous endocrine, reproductive, and metabolic manifestations of the disorder. In Traditional Chinese Medicine (TCM), the tongue is regarded as an external mirror reflecting the state of internal organs and systemic metabolic health. Modern clinical studies have demonstrated that specific tongue phenotypes—such as variations in color, coating thickness, and sublingual vessel morphology—are significantly correlated with endocrine disorders and inflammatory states, including PCOS and insulin resistance (5). Specifically, the digital quantification of tongue features provides objective biomarkers that can bypass the subjectivity of traditional visual inspection, offering a standardized approach for continuous health monitoring.

However, relying solely on tongue images may limit the diagnostic precision due to the multi-systemic complexity of gynecological diseases. To bridge this gap, artificial intelligence and cross-modal feature fusion techniques offer a powerful solution (6). By integrating high-level tongue image features extracted via deep learning with structured clinical data, AI models can capture the intricate relationships between external physical signs and internal physiological status. This cross-modal synergy not only enhances diagnostic accuracy but also aligns with the principles of personalized medicine and chronic disease management. In this study, we developed a multimodal clinical screening framework that integrates digitized tongue-image phenotypes with structured demographic, anthropometric, reproductive, endocrine, and metabolic variables. The framework was designed to support PCOS risk stratification as an adjunct to routine clinical assessment.

## 2 Methods

### 2.1 Framework architecture

**Figure 1:**
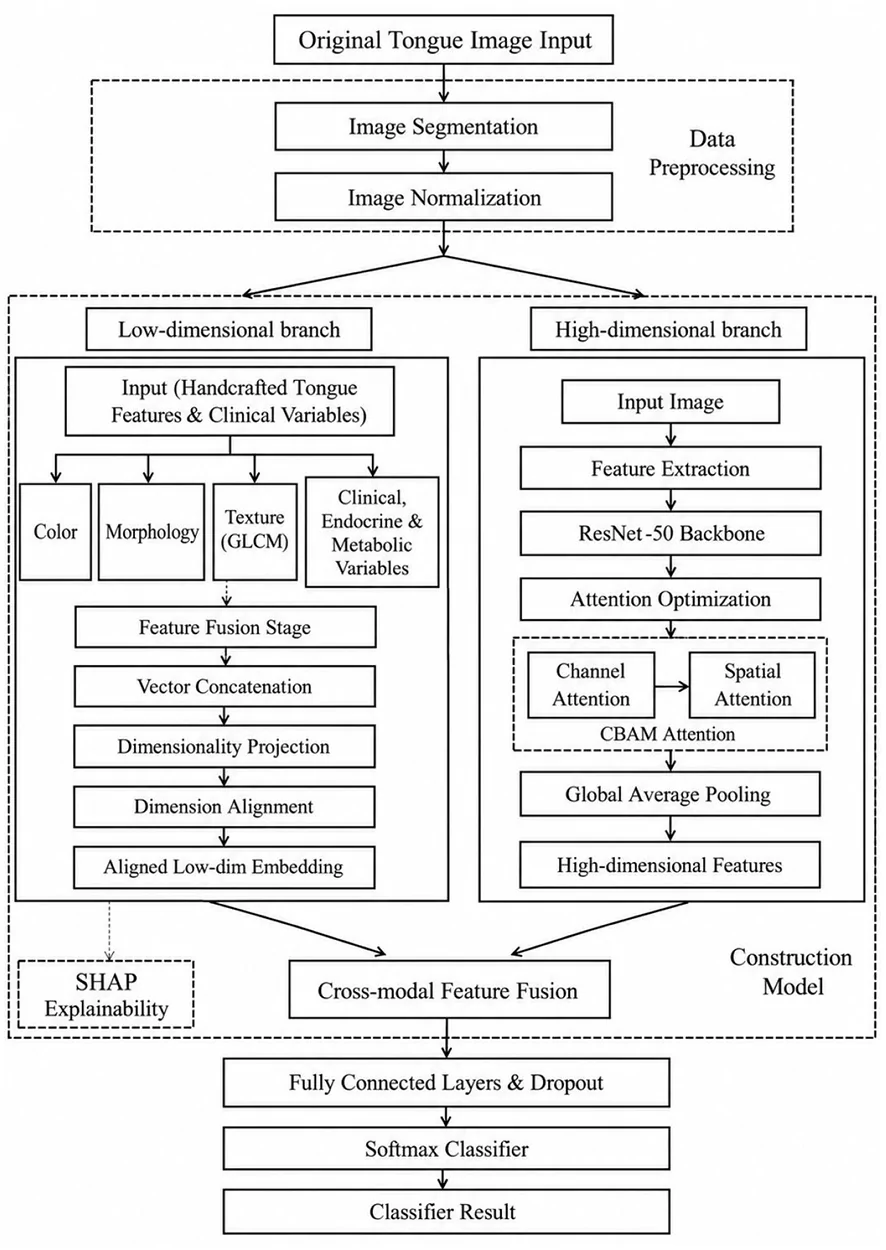
Overall framework.

The diagnostic framework for PCOS is architected upon a multi-modal information fusion paradigm that systematically integrates hand-crafted lingual phenotypes with deep-encoded semantic features. As illustrated in the Figure 1, the system initiates with a dual-branch parallel feature extraction process: a low-dimensional branch processes prespecified demographic, anthropometric, reproductive, endocrine, metabolic, and handcrafted tongue-image variables, while a high-dimensional branch uses an attention-augmented ResNet-50 backbone to extract deep visual features from tongue images. While a high-dimensional branch leverages an attention-augmented ResNet-50 backbone to capture abstract pathological patterns. To bridge the structural gap between these heterogeneous data sources, both feature sets are projected into a unified latent space for dimensional alignment and synergistically integrated via a cross-modal fusion layer. This comprehensive representation is finally processed by a classification head to achieve an end-to-end prediction of PCOS risk, ensuring that diagnostic decisions are informed by both macroscopic clinical observations and microscopic deep-learning insights.

### 2.2 Research data

In this study, a specialized multimodal dataset was curated for the intelligent diagnosis of Polycystic Ovary Syndrome (PCOS). The primary data were prospectively collected from the Department of Preventive Treatment at the Kunshan Hospital of Integrated Traditional Chinese and Western Medicine. All lingual images and clinical endocrine profiles were acquired in real-time during routine gynecological screenings and outpatient consultations, with diagnoses confirmed by senior clinicians based on standardized protocols. To ensure the statistical validity and robustness of our findings, we performed a prior sample size calculation. Assuming a prevalence of PCOS in the screened population of approximately 15–20% and an expected model sensitivity of 0.85, the minimum required sample size was determined to be 420 cases based on a 95% confidence level and a 5% margin of error. To further enhance the diagnostic accuracy and the generalization ability of the deep learning framework. After rigorous quality control and application of the eligibility criteria, a total of 2,315 participants were included, comprising 856 participants with PCOS and 1,459 controls without a diagnosis of PCOS. PCOS was diagnosed by senior clinicians according to the Rotterdam criteria after the exclusion of relevant alternative endocrine disorders. A diagnosis required at least two of the following three features: (1) ovulatory dysfunction, assessed according to menstrual history; (2) clinical and biochemical hyperandrogenism, evaluated using serum testosterone measurements; and (3) polycystic ovarian morphology, assessed by transabdominal ultrasonography.

Eligible participants were reproductive-age women between 18 and 40 years with complete clinical phenotypic profiles and valid tongue images. Participants in the PCOS group were required to meet the Rotterdam diagnostic criteria, whereas participants in the control group were required not to meet these criteria after clinical assessment. Participants were excluded if they had pregnancy or lactation, other endocrine disorders, severe systemic diseases, lingual surface interference, or inability to cooperate with the standardized tongue image acquisition protocol. Detailed exclusion criteria are provided in the note of Supplementary Table S1. To ensure high-fidelity visual data and reduce acquisition-related bias, tongue images were captured using a specialized medical imaging device under a controlled clinical environment. Participants were instructed to sit upright and extend the tongue naturally without excessive force to avoid shape distortion. Images were acquired under a standard D65 light source, with the camera fixed approximately 25–30 cm from the mouth and the optical axis kept perpendicular to the tongue surface. Images with motion blur, incomplete tongue exposure, severe reflection, obvious illumination artifacts, active oral infection, or visible staining interference were excluded during quality control. This standardized protocol ensured that the extracted visual features were primarily driven by tongue phenotypic characteristics rather than environmental noise. The participant enrollment, exclusion, grouping, and dataset partitioning process is illustrated in Supplementary Figure S1. The detailed baseline characteristics, including demographic distributions and clinical phenotypic parameters of the PCOS and control groups, are summarized in Supplementary Table S1, with statistical methods, exclusion criteria, and related annotations provided in the table note.

Following data acquisition, the dataset was randomly partitioned into a training-development set (80%, *n* = 1,852) and an independent testing set (20%, *n* = 463). Within the training-development set, a 5-fold cross-validation strategy was implemented to optimize hyper-parameters and prevent overfitting. This ensures that the model's performance is validated on unseen data, providing a robust estimate of its clinical utility.

### 2.3 Segmentation of the tongue picture

Given the complex perioral backgrounds and variable lighting in clinical settings, isolating the lingual region of interest (ROI) is a prerequisite for reliable downstream analysis (7). In this study, we established a high-standard segmentation benchmark to eliminate extraneous anatomical interference. Expert-guided manual annotations were performed on 2,315 clinical images using Labelme (version 5.6.1) to define precise tongue contours.

To achieve optimal mask generation, we implemented a deep-learning pipeline centered on the nnU-Net architecture (8), selected for its superior adaptive learning and class-balancing capabilities in biomedical imaging. The network was initialized using transfer learning from the Carvana dataset and trained at a resolution of 512 × 512 pixels. We employed the Adam optimizer (β_1_= 0.9, β_2_ = 0.99) with a batch size of 32 for 50 epochs. To ensure convergence stability, a cosine annealing scheduler was applied with an initial learning rate of 1 × 104.

The robustness of this approach was validated through a comparative evaluation against several benchmark models, including the Snake model, FCN, and standard U-Net. As detailed in Table 1, the nnU-Net variant demonstrated near-perfect performance across all metrics, achieving a Dice Similarity Coefficient of 0.9579, a sensitivity of 0.9689, and an F1-score of 0.9686. This statistically validated segmentation framework ensures that the subsequent feature fusion is driven exclusively by high-fidelity tongue tissue data.

**Table 1:** Performance comparison of three segmentation algorithms.

Methods	Dice coefficient	Recall	Precision	F1–score
Snake	63.82%	62.3%	65.71%	64.44%
FCN	92.52%	90.52%	90.10%	90.57%
U–net	93.57%	91.20%	91.32%	92.57%
**nnUNet**	**95.79%**	**96.83%**	**96.89%**	**96.86%**

Bold values indicate the best-performing results among the compared methods.

### 2.4 Multi-modal feature extraction

Feature extraction is used both as a term for low-dimensional feature extraction and high-dimensional feature extraction.

#### 2.4.1 Low-dimensional feature integration

The low-dimensional branch incorporated a prespecified set of clinical, laboratory, and handcrafted tongue-image variables that were entered directly into the final prediction model. The clinical component included demographic and anthropometric variables, reproductive and clinical hyperandrogenism-related variables, and serum endocrine and metabolic variables. The laboratory variables entered into the final prediction model were total testosterone, the LH/FSH ratio, and fasting insulin. The handcrafted tongue-image component included color, texture, and morphological descriptors extracted from the segmented tongue region. A complete list of the variables entered into the final model is provided in Table 2. These variables were selected not only for their clinical relevance to PCOS pathophysiology but also for their high accessibility in community-level screening scenarios.

**Table 2:** Specification of low–dimensional feature set.

Category	Feature variables	Count	Description & clinical basis
Clinical profiles	Age, BMI, WHR	3	Assessment of basal metabolism and central obesity via anthropometric metrics.
	Menstrual cycle status	2	Self–reported regularity and mean duration; indicators of ovulatory dysfunction.
	Hyperandrogenism	2	Standardized self–assessment scores for hirsutism and acne.
Endocrine and metabolic profiles	Total testosterone (TT), LH/FSH ratio, fasting insulin (FINS)	3	Serum endocrine and metabolic variables associated with hyperandrogenism, hypothalamic–pituitary–ovarian axis function, and insulin resistance.
Color moments	CIELAB moments (L^*^, a^*^, b^*^ )	3	1st−3rd moments; quantifies lingual perfusion and systemic thermal status.
	HSV moments (*H, S, V*)	3	1st−3rd moments; captures chromatic saturation and brightness variations.
	YCrCb moments (*Y, Cr, Cb*)	3	1st−3rd moments; decouples luminance to mitigate uneven illumination bias.
Texture (GLCM)	Contrast, correlation	2	Quantifies local intensity variations and linear dependency of gray levels.
	Energy, homogeneity	2	Measures textural orderliness and smoothness; differentiates coating thickness.
	Entropy, dissimilarity	2	Reflects textural complexity and local contrast associated with “viscid” coating.
Morphology	Shape symmetry index	1	Geometric ratio of bilateral tongue areas to evaluate structural balance.
	Fissure density	1	Quantitative ratio of fissure pixels to total lingual area; linked to chronic inflammation.
	Area/Perimeter ratio	1	Compactness metric to quantify lingual edema or “teeth–marked” phenotypes.
Total		28	

The symbol “*” is part of the standard CIELAB color notation (L, a, and b) and does not indicate statistical significance.

To augment these foundational metrics, we extracted a suite of Digital Lingual Phenotypes that serve as objective surrogates for systemic metabolic signatures (9). This phenotypic ensemble comprises Color Moments (mean, variance, and skewness) in the CIELAB color space, which quantify systemic circulatory status, and Gray-Level Co-occurrence Matrix (GLCM) descriptors (Contrast, Correlation, Energy, and Homogeneity), which characterize the micro-textural properties of the tongue coating. Together with morphological traits such as tongue shape and fissure presence, these features are concatenated into a heterogeneous low-dimensional vector. To ensure effective cross-modal synergy, this vector is subsequently processed through a Dimensional Alignment Layer, utilizing an MLP projection head to map these discrete clinical and manual features into a 128–dimensional latent space, consistent with the deep visual embeddings.

The extraction of low-dimensional features was performed through an automated image-processing pipeline (10, 11). Following the precise segmentation of the lingual region, the isolated tongue masks were utilized to crop the regions of interest (ROI) from the original clinical images. Specifically, pixel-level statistical analysis was conducted to compute color and texture descriptors, while morphological contours were derived from the binary mask geometry. Through this process, we established an expanded feature set comprising 28 distinct variables across four primary domains. The Clinical Demographic Profile was refined beyond baseline metrics to include indicators such as Waist-to-Hip Ratio (WHR), menstrual cycle duration, and irregularity severity, providing a nuanced physiological context. Concurrently, the Digital Tongue Phenotypes were systematically deconstructed:

(1) Chromatic Distribution, featuring 9 parameters derived from the first three moments of CIELAB and HSV color spaces to capture subtle cyanotic or inflammatory shifts.
(2) Micro-textural Descriptors, utilizing 6 core Gray-Level Co-occurrence Matrix (GLCM) parameters—Contrast, Correlation, Energy, Homogeneity, Entropy, and Dissimilarity—to quantify coating thickness and viscidity.
(3) Morphological Traits, incorporating quantitative metrics for tongue symmetry, area-to-perimeter ratio, and fissure density. To mitigate feature redundancy and numerical instability.

All 28 variables underwent Z-score standardization prior to being mapped into the 128-dimensional latent space via the MLP encoder. The comprehensive definitions, computational methods, and clinical significance of these variables are summarized in Table 2.

Following the feature extraction, a significant challenge arises from the structural heterogeneity between the sparse clinical-phenotypic vector ($V_{raw}\;\in {\mathbb{R}}^{28}$) and the high-dimensional visual features. To facilitate the subsequent integration with deep visual embeddings, we implemented a Dimensional Alignment Layer to map the heterogeneous low-dimensional data into a standardized latent space. Twenty Eight raw features undergo Z-score normalization to eliminate scale-related bias. These normalized inputs are then processed through a Low-dimensional Projection Head, consisting of a three-layer Multi-Layer Perceptron (MLP). This module is specifically designed to transform the discrete clinical metrics and handcrafted lingual descriptors into a 128-dimensional dense representation, denoted as $V_{low}\;\in {\mathbb{R}}^{128}$ . This mapping process is crucial for cross-modal fusion, as it ensures that the subsequent integration stage operates on a balanced representation space. By calibrating both modalities to an equivalent scale and dimension, we prevent the model from being biased toward the high-dimensional visual backbone, thereby enabling a truly synergistic synthesis of objective clinical indicators and deep-encoded lingual patterns.

#### 2.4.2 High-dimensional feature integration

To extract discriminative visual representations from complex lingual surfaces, we developed a high-dimensional feature integration branch based on the ResNet-50 backbone (12) augmented with a Convolutional Block Attention Module (CBAM). The primary objective of this branch is to transform raw pixel data into high-level semantic embeddings that capture subtle pathological indicators, such as localized congestion, coating hypertrophy, or fissured topography (13). By integrating the attention mechanism (14) directly into the residual blocks, the framework can autonomously prioritize informative regions and suppress non-diagnostic background noise.

The core of our visual branch is the ResNet-50 architecture, which utilizes bottleneck residual blocks to facilitate the training of deep networks while mitigating the vanishing gradient problem. We initialize the backbone with pre-trained ImageNet weights to leverage established low-level visual filters. The output of a standard residual block before activation can be expressed as Equation (1):


$$\begin{array}{c} y\;=F\left(x,\left\{W_{i}\right\}\right)\;+\;x \end{array}$$ (1)

where *x* is the input vector, *y* is the output vector, and the function $F\left(x,\left\{W_{i}\right\}\right)$ represents the residual mapping to be learned, typically consisting of a series of 1 × 1, 3 × 3, and 1 × 1 convolutions in the bottleneck architecture. This formulation ensures that if the identity mapping is optimal, the solver can simply drive the weights of the multiple non-linear layers toward zero to approach the identity mapping. In the context of PCOS tongue image analysis, this structural prior allows the model to preserve low-level textural gradients while extracting high-level pathological semantics through 50 layers of transformation.

While the residual mechanism excels at maintaining signal integrity in deep architectures, standard ResNet-50 treats all spatial regions and channel-wise features with equal importance. In clinical tongue diagnosis, however, diagnostic information is often non-uniformly distributed. For instance, subtle chromatic shifts indicative of PCOS may only appear in specific lingual zones, or certain feature channels may capture redundant background noise from varying illumination conditions.

**Figure 2:**
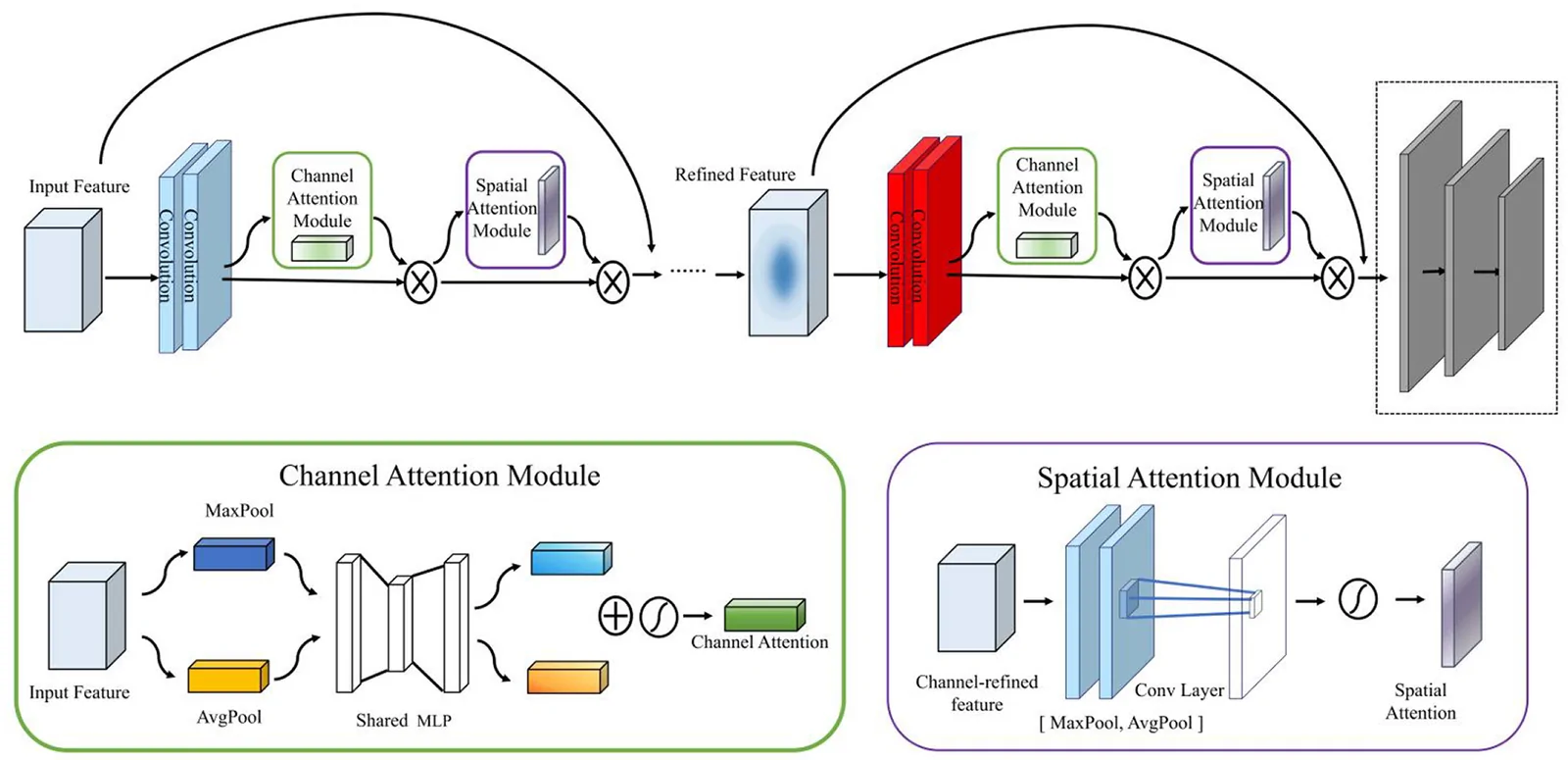
Specification of CBAM Mechanism.

To overcome this limitation and enable the model to selectively focus on the most informative “what” and “where,” we integrate a CBAM into the residual stages. This module functions as an adaptive feature refiner that re-weights the feature maps through a sequential dual-attention process, as illustrated in Figure 2.

As illustrated in Figure 2, the CBAM sequentially infers attention maps along two independent dimensions—channel and spatial—to refine the input feature map *F* ∈ ℝ^{*H*×*W*×*C*}^. The refined feature map *F*″ is computed through the following dual-stage process: Channel Attention Module (*M*_*c*_) and Spatial Attention Module (*M*_*s*_).

Channel Attention Module (*M*_*c*_) prioritizes “what” lingual features are diagnostic by exploiting the inter-channel relationships. To aggregate spatial information, both Average Pooling and Max Pooling are applied simultaneously. The first stage involves spatial information aggregation, where the input feature map *F* ∈ ℝ^{*C*×*H*×*W*}^ is compressed along its spatial dimensions. Unlike conventional methods that rely solely on average pooling, we utilize both Global Average Pooling (GAP) and Global Max Pooling (GMP) to produce two distinct channel-wise descriptors. As shown in (2), (3), these descriptors, denoted as $F_{avg}^{c}$ and $F_{\max}^{c}$, capture the global distribution and the most salient signals of the lingual features.


$$\begin{array}{c} F_{avg}^{c}\;=\;AvgPool\left(F\right) \end{array}$$ (2)

$$\begin{array}{c} F_{\max}^{c}\;=MaxPool\left(F\right) \end{array}$$ (3)

where *AvgPool*(•) and *MaxPool*(•) denote the spatial aggregation operations that collapse the $H times W$ dimensions into a 1 × 1 spatial extent for each of the C channels.

In the second stage, these descriptors are fed into a Shared Multi-Layer Perceptron (MLP) to perform non-linear mapping and cross-channel interaction. The MLP architecture contains one hidden layer with a reduction ratio *r* to minimize parameter overhead. The transformation process is described in Equation (4):


$$\begin{array}{c} f_{avg}^{c}\;=\;W_{1}\left(W_{0}\left(F_{avg}^{c}\right)\;\right)\;;\;f_{\max}^{c}\;=W_{1}\left(W_{0}\left(F_{\max}^{c}\right)\;\right) \end{array}$$ (4)

where $W_{0\;}\in \;{\mathbb{R}}^{C/r\;\times C}$ and $W_{1\;}\in \;{\mathbb{R}}^{C\times C/r\;}$ represent the shared weights of the MLP Here, *W*_0_acts as a dimensionality reduction layer, while *W*_1_ restores the vector to the original channel dimension C. By utilizing shared weights, the network ensures that the learned semantic importance remains consistent across different pooling perspectives. The final stage concludes with feature fusion and activation to generate the channel-wise attention weights. As illustrated in Equation (5), the outputs $f_{avg}$ and $f_{\max}$ are merged via element-wise summation, and the resulting vector is passed through a Sigmoid activation function to produce the final attention map *M*_*c*_:


$$\begin{array}{c} M_{c}\left(F\right)\;=\;\sigma \left(f_{avg}\;+f_{\max}\right) \end{array}$$ (5)

where σ denotes the sigmoid function, ensuring that each weight in $M_{c\;}\in \;{\mathbb{R}}^{C\times 1\times 1}$ is bounded between 0 and 1. This map is then multiplied element-wise with the original feature map *F* to obtain the refined intermediate representation *F*′, effectively rescaling the importance of each channel according to its diagnostic relevance for PCOS.

Complementary to the channel attention branch, the Spatial Attention Module *M*_*s*_focuses on identifying “where” the most discriminative pathological markers are located on the lingual surface. This module takes the intermediate feature map *F*′, refined by the channel branch, as its input. To aggregate spatial contexts, the module first applies pooling operations along the channel axis to generate two 2D maps. As shown in Equations (6) and (7), these maps represent the average-pooled and max-pooled features across all channels:


$$\begin{array}{c} F_{avg}^{s}\;=\;AvgPool_{chan}\left(F^{\prime }\right) \end{array}$$ (6)

$$\begin{array}{c} F_{\max}^{s}\;=MaxPool_{chan}\left(F^{\prime }\right) \end{array}$$ (7)

where *AvgPool*_*chan*_(•) and *MaxPool*_*chan*_(•) denote the pooling operations across the channel dimension, resulting in two spatial descriptors $F_{avg}^{s}\;\in \;{\mathbb{R}}^{1\;\times \;H\;\times \;W}$ and $F_{\max}^{s}\;\in \;{\mathbb{R}}^{1\;\times \;H\;\times \;W}$. This step effectively summarizes the feature responses at each spatial location, highlighting regions with consistent or peak intensity.

In the second stage, these two descriptors are concatenated to form a composite feature descriptor that captures a rich spatial context. This concatenated map is then processed by a standard convolutional layer to produce the raw spatial attention scores. As described in Equation (8), a large receptive field is utilized to capture broader contextual information:


$$\begin{array}{c} M_{s\;}\left(F^{\prime }\right)\;=\;\sigma \left(f^{7\times 7}\left(\left[F_{avg}^{s};F_{\max}^{s}\right]\right)\right) \end{array}$$ (8)

where σ denotes the sigmoid activation function, and *f*^7 × 7^ represents a convolution operation with a filter size of 7 × 7. The use of a relatively large kernel ensures that the module can perceive the spatial relationships between distant lingual regions, such as the correlation between the tongue tip and the root. The sigmoid function maps the output to a normalized range of [0, 1], creating a spatial mask that indicates the relative importance of each pixel.

The final stage involves the calibration of the feature map based on the computed spatial weights. The refined feature map *F*″ is obtained by performing an element-wise multiplication between the spatial attention map *M*_*s*_ and the intermediate map *F*′. This process ensures that the network selectively enhances pixels corresponding to diagnostic features—such as specific coating textures or fissured patterns—while suppressing irrelevant background regions. The output *F*″ thus represents a highly refined high-dimensional embedding that encapsulates both channel-wise semantic priorities and spatial-wise geometric importance, providing a robust foundation for the subsequent classification decision. To transform this high-dimensional tensor into a vector suitable for multimodal fusion, we apply a Global Average Pooling (GAP) operation. As shown in Equation (9), this operation computes the spatial average of each feature map to produce a robust global descriptor $V_{raw\text{\_}visual}\in R^{2048}$:


$$\begin{array}{c} V_{raw\text{\_}visual}\;=\;GAP\left(F^{\prime \prime }\right) \end{array}$$ (9)

where *GAP*(•) reduces the *C* × *H* × *W* dimensions by averaging all pixel values within each channel, thereby ensuring translation invariance and reducing the risk of overfitting.

The integration of the CBAM into our ResNet50 backbone was strategically motivated by the dual-dimensional nature of tongue pathological features. Unlike traditional Squeeze-and-Excitation (SE) blocks that focus exclusively on inter-channel relationships, CBAM sequentially infers attention maps along two separate dimensions: channel and space. In the context of PCOS tongue diagnosis, channel attention enables the model to prioritize critical color-texture signatures, while spatial attention assists in locating regional lesions. This dual-attention mechanism effectively suppresses background noise and emphasizes diagnostically relevant regions, providing a more robust visual feature representation than global pooling or channel-only attention methods.

Furthermore, the superiority of CBAM over other attention mechanisms lies in its “fine-grained heuristic” approach to feature recalibration. While global attention mechanisms like the Non-local Network effectively capture long-range dependencies, they often introduce excessive computational overhead and may dilute localized pathological signals due to over-smoothing. CBAM, through its sequential architecture of Channel and Spatial modules, achieves a superior balance between global context and local precision. By first identifying “what” is important through channel inter-dependencies and subsequently “where” is important through spatial mapping, CBAM acts as a robust feature refiner that is more computationally efficient than Transformers while providing more task-specific focus than the Squeeze-and-Excitation (SE) block. This refined focus is particularly advantageous for PCOS diagnosis, where the pathological evidence is often distributed across heterogeneous scales and requires a synergistic capture of both holistic tongue conditions and localized morphological changes.

To bridge the gap between the high-dimensional visual branch and the clinical metadata, this 2,048-dimensional vector is subsequently passed through a linear projection layer. This final step maps the visual information into a 128-dimensional deep visual embedding (*V*_*deep*_). This integration ensures that the most discriminative visual cues—refined by the attention mechanism—are preserved in a compact latent space, structurally prepared for the subsequent synergistic alignment with the handcrafted clinical-phenotypic features.

### 2.5 Cross-modal feature fusion

The objective of cross-modal fusion is to integrate the global visual semantics extracted from tongue images with the granular clinical and tongue phenotypic evidence (24). To achieve effective interaction between heterogeneous modalities, we propose an attention-guided cross-modal feature fusion module. Specifically, the 128-dimensional deep visual embedding *V*_*deep*_, extracted from the ResNet50-CBAM branch, and the 128-dimensional low-dimensional clinical-phenotypic embedding *V*_*low*_, obtained from clinical profiles and handcrafted tongue features, are first concatenated to form a unified multimodal representation in Equation (10):


$$\begin{array}{c} V_{cat}\;=\left[\;V_{deep}\;;V_{low}\;\right],\;V_{cat}\in \;{\mathbb{R}}^{256} \end{array}$$ (10)

where [;] denotes vector concatenation. Although concatenation provides a direct way to combine multimodal features, it treats all feature dimensions equally and may fail to distinguish diagnostically important information from redundant signals. Therefore, a fusion-level attention mechanism is further introduced to adaptively recalibrate the concatenated representation, details are shown in (11), (12).


$$\begin{array}{c} A_{fusion\;}=\;\sigma \left(W_{2}\delta \left(W_{1}V_{cat}\;+\;b_{1}\right)+b_{2}\right) \end{array}$$ (11)

$$\begin{array}{c} V_{fusion}=A_{fusion\;}⊙\;V_{cat} \end{array}$$ (12)

where δ represents the ReLU activation function, σ denotes the sigmoid function, $A_{fusion}\in \;{\mathbb{R}}^{256}$ is the learned attention weight vector, and ⊙ denotes element-wise multiplication. Through this mechanism, the model can assign higher weights to diagnostically informative visual or clinical-phenotypic dimensions while suppressing less relevant or redundant features.

This attention-guided fusion strategy is particularly suitable for tongue-image-based PCOS screening because the diagnostic contribution of different modalities may vary across individuals. For example, tongue color and texture features may provide useful external phenotypic cues, whereas menstrual status, BMI, and endocrine-related clinical variables may reflect internal physiological abnormalities more directly. By adaptively integrating these complementary features, the proposed fusion module enables a more flexible and clinically meaningful representation for PCOS risk prediction.

### 2.6 Loss function optimization

The selection of an appropriate objective function is pivotal for effectively training the multimodal network, particularly when facing significant class imbalance in clinical datasets. In this study, we evaluated and compared several loss functions to determine the optimal strategy for PCOS diagnosis. The specific mathematical formulations and the comparative framework of these functions are summarized in Table 3.

**Table 3:** Mathematical formulations of the loss functions used in this study.

Loss function	Specific formula
Cross–entropy loss	$\begin{array}{c} L_{CE}\;=\;-\;\frac{1}{N}\overset{N}{\underset{i\;=\;1}{\sum }}log\left(p_{i,y_{i}}\right)\;\left(13\right) \end{array}$
Focal loss	$\begin{array}{c} L_{FL}\;=\;-\;\frac{1}{N}\;\overset{N}{\underset{i\;=\;1}{\sum }}w_{y_{i\;}}{\left(1\;-\;p_{i,y_{i\;}}\right)}^{\gamma }log\left(p_{i,y_{i\;}}\right)\left(14\right) \end{array}$
LDAM loss	$\begin{array}{l} L_{LDAM}=-\frac{1}{N}\sum_{i=1}^{N}\log(\frac{\exp\left(z_{i},y_{i}-\Delta y_{i}\right)}{\exp\left(z_{i},y_{i}-\Delta y_{i}\right)+\sum_{j\neq y_{i}}\exp(z_{i,j})})\\ \;where\;\Delta_{j}=\frac{c_{m}}{n_{j}^{\frac{1}{4}}}\left(15\right) \end{array}$
LMF loss	$\begin{array}{c} L_{LMF}\;=\lambda \;L_{FL\;}+\left(1\;-\;\lambda \right)\;L_{LDAM}\left(16\right) \end{array}$

For the above equations, *N* denotes the number of training samples, *y*_*i*_denotes the ground-truth label of the i-th sample, *z*_*i, j*_ denotes the output logit of class *j*, and *p*_*i*,_*y*__*i*__represents the predicted probability of the true class after softmax normalization. *w*_*y*_*i*__is the class-specific weighting factor used to reduce the influence of class imbalance, and γ is the focusing parameter in Focal loss. In LDAM loss, *n*_*j*_ denotes the number of training samples in class j, *C*_*m*_ is the margin scaling constant, and Δ_*j*_ represents the class-dependent margin. The proposed LMF loss combines the hard-sample focusing ability of Focal loss with the margin-based class separation ability of LDAM loss. The coefficient λ controls the relative contribution of the two components (26).

Initially, the standard Cross-Entropy loss was considered as the baseline objective function, as shown in Equation (13). However, Cross-Entropy loss assigns equal importance to all samples, which may lead the model to be biased toward the majority class in imbalanced clinical datasets. To alleviate this issue, Focal loss was introduced to down-weight well-classified easy samples and force the model to focus more on difficult or boundary cases, as shown in Equation (14). In addition, LDAM loss was adopted to introduce a larger decision margin for classes with fewer samples, thereby improving class separability in the latent feature space, shown in Equation (15). By combining these two strategies, the proposed LMF loss is designed to improve the sensitivity and robustness of PCOS detection, especially for samples that are difficult to distinguish using standard classification objectives, which is shown in Equation (16).

To ensure experimental rigor and reproducibility, all hyperparameters of the LMF loss were fixed before final model evaluation and were kept identical across all loss-function comparison experiments. These parameters were determined according to the class distribution of the study cohort and commonly adopted settings in Focal loss and LDAM loss, rather than being adjusted according to the independent test-set performance. Specifically, the focusing parameter of Focal loss was set to γ = 2.0, which is a standard setting used to reduce the contribution of well-classified samples and emphasize difficult cases. To compensate for class imbalance, class-specific weights were calculated using inverse class frequency, defined as *w*_*j*_ = $\frac{N}{\left(Kn_{j}\right)}$, where *N* denotes the total number of samples, *K* denotes the number of classes, and *n*_*j*_ denotes the number of samples in class *j*. Based on the class distribution of the enrolled cohort, the weights were set to 0.793 for the control class and 1.352 for the PCOS class. For LDAM loss, the maximum margin was set to *m*_*max*_ = 0.5, and the class-dependent margin was calculated as $\Delta_{j}\;=\frac{m_{\max}\left(n_{j}^{\frac{-1}{4}}\right)}{m_{\max}{(n}_{k}^{\frac{-1}{4}})}$. Accordingly, the margins were 0.438 for the control class and 0.500 for the PCOS class, assigning a larger decision margin to the minority PCOS class. The balancing coefficient of the LMF loss was set to λ = 0.5, giving equal importance to the hard-sample focusing effect of Focal loss and the margin-based class separation effect of LDAM loss. These settings were consistently used throughout the experiments to improve the transparency and reproducibility of the proposed loss-function optimization strategy.

### 2.7 Training protocol and implementation details

The proposed multimodal framework was implemented using the PyTorch deep learning library and executed on an Ubuntu 20.04 infrastructure equipped with an NVIDIA Tesla V100-PCIe (32GB) GPU. To ensure efficient convergence and numerical stability, we utilized the Adam optimizer with momentum parameters set to β_1_ = 0.9 and β_2_ = 0.999. The initial learning rate was established at 3 × 10^−3^. To prevent the model from becoming trapped in local optima and to refine the weights in the later stages of training, we employed a Cosine Annealing scheduler. This strategy dynamically adjusted the learning rate over 100 epochs, decaying it toward a minimum threshold of 3 × 10^−8^.

To compensate for the relatively specialized nature of medical tongue images and to accelerate the feature extraction process, a Transfer Learning strategy was adopted. The ResNet-50 backbone was initialized with weights pre-trained on the ImageNet dataset, allowing the model to leverage robust low-level visual primitives. Furthermore, to enhance the model's generalization capability and mitigate the risk of overfitting, extensive data augmentation was performed during the training phase, including random scaling, horizontal flipping, and rotation. The mini-batch size was fixed at 32. Throughout the iterative training process, the model's performance was monitored on a longitudinal validation set, and the specific version yielding the highest Area Under the Curve (AUC) was preserved as the final diagnostic tool for PCOS.

To further improve the transparency and reproducibility of the experimental design, we summarized the implementation settings, training hyperparameters, validation strategy, and model comparison protocol in Table 4. All baseline models, ablation models, and the proposed multimodal framework were trained and evaluated under the same data partition, preprocessing procedure, optimization strategy, and evaluation metrics unless otherwise specified. The independent test set was used only for final model evaluation and was not involved in model training, hyperparameter selection, or checkpoint selection.

**Table 4:** Implementation details and experimental settings for reproducibility.

Item	Settings
Framework	PyTorch
Operating system	Ubuntu 20.04
Hardware	NVIDIA Tesla V100-PCIe GPU, 32 GB memory
Optimizer	Adam
Adam parameters	β_1_ = 0.9 and β_2_ = 0.999
Learning rate	$lr_{\max}:\;3\;\times \;10^{-3}\;$
	$lr_{\min}:\;3\;\times \;10^{-8}$
Learning-rate scheduler	Cosine annealing
Training epochs	100
Mini-batch size	32
Evaluation metrics	Accuracy, AUC, Precision, Recall, Specificity, and F1-score
Loss-function comparison	CE loss, Focal loss, LDAM loss, and LMF loss were compared under the same training protocol
LMF loss parameters	γ = 2.0, *m*_max_ = 0.5, λ = 0.5 with class weights calculated by inverse class frequency

## 3 Results

### 3.1 Evaluation indices

To rigorously benchmark the diagnostic efficacy of the proposed multimodal fusion model, we utilized a comprehensive suite of statistical metrics derived from the confusion matrix. In the context of PCOS screening, the model's predictions are categorized into four fundamental outcomes: True Positives (TP) and True Negatives (TN) represent accurate identifications of pathological and healthy states, respectively; whereas False Positives (FP) and False Negatives (FN) denote misdiagnosis and missed detections. Based on these primary data points, the following high-level indicators were computed:

Accuracy (ACC) reflects the overall proportion of correct classifications across the entire experimental cohort, serving as a primary measure of predictive reliability.


$$\begin{array}{c} ACC=\;\frac{TP+TN}{TP+FN+FP+TN} \end{array}$$ (17)

Precision quantifies the model's ability to minimize “false alarms” by calculating the ratio of true PCOS cases among all predicted positives.


$$\begin{array}{c} Precision=\;\frac{TP}{TP+FP} \end{array}$$ (18)

Sensitivity measures the detection rate of actual PCOS patients. Higher sensitivity is paramount in clinical settings to ensure that early-stage pathological features are not overlooked.


$$\begin{array}{c} Recall=\;\frac{TP}{TP+FN} \end{array}$$ (19)

Specificity specifically evaluates the framework's robustness in correctly excluding non-PCOS individuals, which is critical for reducing unnecessary clinical interventions.


$$\begin{array}{c} Specificity=\;\frac{TN}{TN+FP} \end{array}$$ (20)

The F1 score accurately provides a harmonic balance between precision and sensitivity, offering a more equitable assessment of performance on our class-imbalanced dataset.


$$\begin{array}{c} F1=\;\frac{2\times TPR\times PPV}{TPR+PPV} \end{array}$$ (21)

Furthermore, we employed the Receiver Operating Characteristic (ROC) curve to visualize the dynamic trade-off between the True Positive Rate (TPR) and the False Positive Rate (FPR). The Area Under the Curve (AUC) was utilized as the definitive metric to summarize the model's discriminative capacity; an AUC value approaching 1.0 indicates near-perfect separation between PCOS and control groups in the latent feature space (15).

### 3.2 Diagnostic superiority of multimodal fusion strategy

In this section, we systematically evaluate the contribution of multimodal data integration to the diagnostic performance of PCOS (16). To ensure a rigorous and unbiased assessment, the dataset was partitioned into a training set and an independent testing set with a ratio of 8:2. During the training phase, a 5-fold cross-validation strategy was implemented to optimize hyperparameters and mitigate the risk of overfitting. To establish a robust baseline, we compared three established unimodal deep learning architectures—AlexNet, VGGNet, and ResNet50—with our proposed multimodal framework. The fusion strategy technically bridges the gap between heterogeneous data sources by concatenating high-level visual features (extracted via the ResNet50-CBAM backbone) with 28 critical clinical phenotypic parameters. All models were evaluated on the same independent testing set (*N* = 463) using standard clinical metrics, including ACC, AUC, Precision, Recall, and F1-score, to provide a comprehensive benchmark for clinical screening application (17).

**Figure 3:**
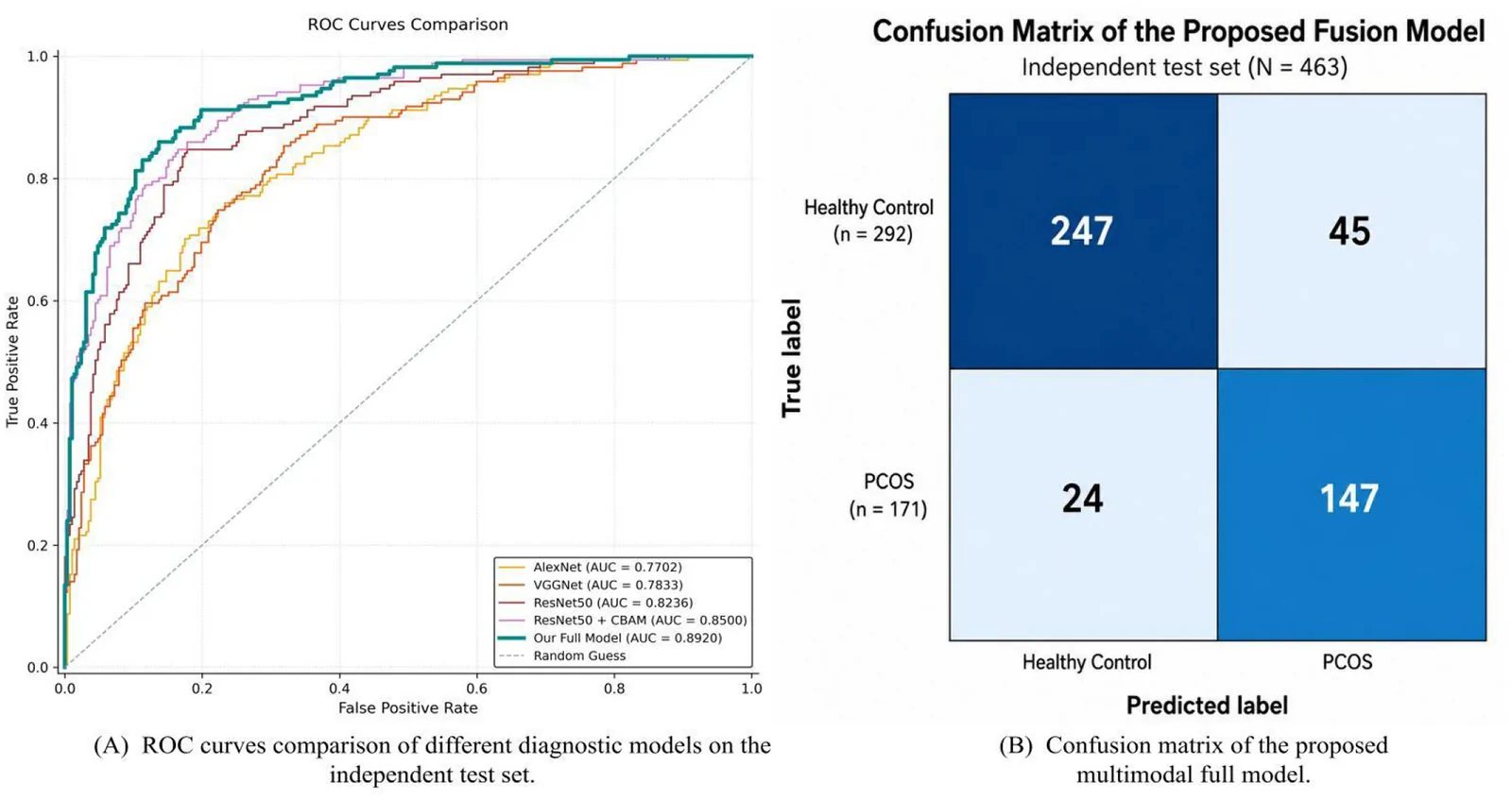
Evaluation of diagnostic performance on the independent test set. **(A)** ROC curves comparison of different diagnostic models on the independent test set. **(B)** Confusion matrix of the proposed multimodal full model.

The comprehensive performance metrics are detailed in Table 5, and the corresponding visual evaluations are presented in Figure 3. Figure 3A displays the ROC curves of the compared models, while Figure 3B illustrates the confusion matrix for our integrated framework.

**Table 5:** Comprehensive performance metrics.

Deep network	ACC (%)	AUC (%)	Precision (%)	F1–score (%)	Recall (%)
AlexNet	72.31 [68.23–76.39]	77.02 [72.36–81.68]	72.34 [68.26–76.42]	72.20 [68.12–76.28]	72.28 [68.20–76.36]
VGGNet	74.36 [70.38–78.34]	78.33 [73.77–82.89]	75.33 [71.40–79.26]	75.55 [71.64–79.46]	75.44 [71.52–79.36]
ResNet50	78.02 [74.25–81.79]	82.36 [78.16–86.56]	77.69 [73.89–81.49]	78.34 [74.59–82.09]	78.01 [74.24–81.78]
ResNet50 + CBAM	80.50 [76.89–84.11]	85.00 [81.08–88.92]	80.20 [76.57–83.83]	80.70 [77.11–84.29]	80.45 [76.84–84.06]
**Fusion model**	**85.10 [81.97–88.43]**	**89.20 [85.81–92.59]**	76.56 [74.19–78.61]	**80.99 [78.51–82.87]**	**85.96 [83.29–89.51]**

All performance metrics were calculated on the independent test set. Values are presented as point estimates with 95% confidence intervals in brackets. The 95% confidence intervals were estimated using bootstrap resampling with 1,000 iterations. AUC was calculated from predicted probabilities, whereas ACC, Precision, F1-score, and Recall were calculated from classification results under the same evaluation protocol. Recall refers to the detection rate of PCOS cases. The best-performing values are shown in bold.

The quantitative results in Table 5 reveal a significant performance disparity between unimodal and multimodal approaches. Unimodal models relying exclusively on tongue imagery, such as the ResNet50-CBAM, achieved an AUC of 0.85 and an Accuracy of 81.64%. Despite the integration of attention mechanisms to refine visual feature extraction, these models encountered a “performance ceiling.” From a clinical perspective, this limitation stems from the fact that PCOS is a systemic endocrine disorder; while tongue manifestations provide a macroscopic reflection of the internal environment, they may lack the granularity required to distinguish complex metabolic nuances when analyzed in isolation.

Conversely, the implementation of the multimodal fusion strategy (FusionBaseline) catalyzed a substantial leap in diagnostic precision. The final multimodal model achieved an accuracy of 85.10%, an AUC of 0.8920, a precision of 76.56%, an F1-score of 80.99%, and a recall of 85.96%. The model correctly identified 147 of 171 participants with PCOS. Its specificity was 84.59%, with 247 of 292 control participants correctly classified, as visualized in the confusion matrix (Figure 3B). Furthermore, a high Specificity of 84.59% was maintained, effectively minimizing false alarms in the healthy control group (*N* = 292). This multi-dimensional improvement proves that the integration of clinical indicators—such as hormonal profiles and metabolic indices—complements the visual evidence from tongue images, allowing the model to capture a more comprehensive pathological signature of PCOS.

### 3.3 Results of different loss function

In clinical practice, the primary objective of an AI-assisted screening tool is to minimize “missed diagnoses” (False Negatives), as undetected PCOS can lead to severe long-term sequelae, including infertility, metabolic syndrome, and endometrial hyperplasia (18). Consequently, our model design prioritizes the detection of True Positives to ensure that the vast majority of patients with potential pathological signatures are identified for further clinical evaluation. While this focus may introduce a manageable rate of False Positives, such a trade-off is clinically acceptable—and often preferred—within a screening context. It establishes a effectively diagnostic method where the cost of a follow-up confirmatory test for a false positive is significantly lower than the personal and socioeconomic burden incurred by a missed diagnosis.

**Figure 4:**
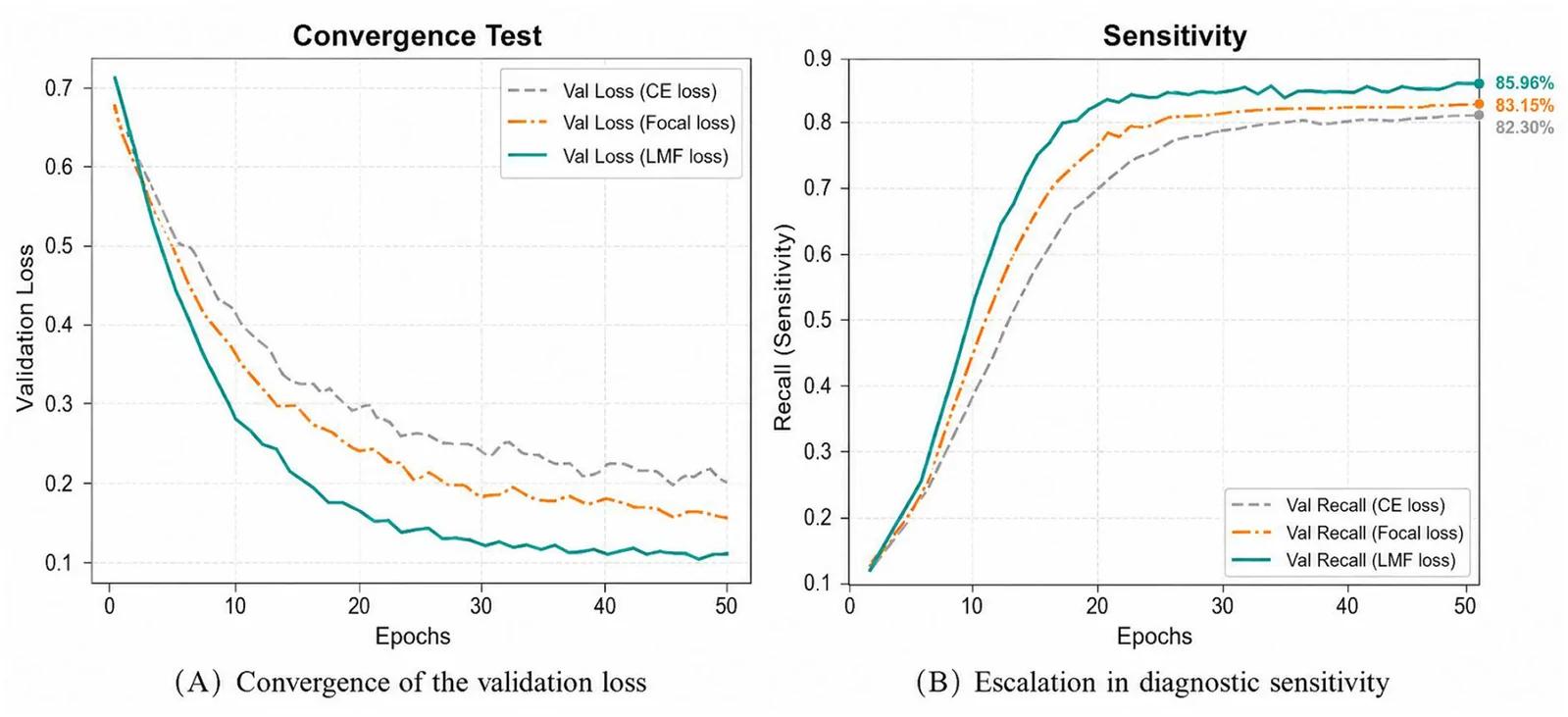
Comparative analysis of training dynamics across different loss functions. **(A)** Convergence of the validation loss. **(B)** Escalation in diagnostic sensitivity.

The choice of loss function is pivotal in aligning the model's objective with clinical priorities. In PCOS screening, the cost of a False Negative significantly outweighs that of a False Positive due to the risk of long-term sequelae. To address this, we conducted an ablation study comparing the standard Cross-Entropy (CE) loss, Focal Loss, and our proposed LMF loss. This experiment aims to evaluate how different loss formulations recalibrate the decision boundary to prioritize high sensitivity in a clinical setting. To further elucidate the optimization efficiency of the proposed LMF loss, we visualized the training dynamics on the validation set over 50 epochs. Figure 4 illustrates the comparative trajectories of the three evaluated loss functions: Cross-Entropy (CE), Focal Loss, and our LMF loss. Specifically, Figure 4A presents the convergence of the validation loss, while Figure 4B displays the corresponding escalation in diagnostic sensitivity.

As depicted in the convergence curves, all three models exhibit a steady decline in validation loss, confirming a stable learning process. However, the LMF loss achieves a distinctively faster and deeper convergence compared to the CE and Focal Loss baselines. By effectively penalizing hard-to-classify samples through the logit-margin term, the LMF loss enables the model to reach a lower global minimum, indicating a more precise fit to the underlying pathological patterns of PCOS.

The superiority of the LMF loss is most pronounced in the sensitivity escalation curves (Figure 4B). While the CE and Focal Loss models encounter performance bottlenecks early in the training phase, the LMF loss facilitates a rapid and robust ascent in Recall, ultimately stabilizing at 85.96%. This represents a significant margin over the Focal Loss (83.15%) and CE (82.30%) models. This trend suggests that the integration of margin-based optimization actively compels the network to focus on boundary cases, significantly reducing the risk of missed diagnosis (False Negatives). Such dynamic efficiency underscores the reliability of the proposed framework as a clinical decision support tool, where high sensitivity is paramount for effective population screening.

**Figure 5:**
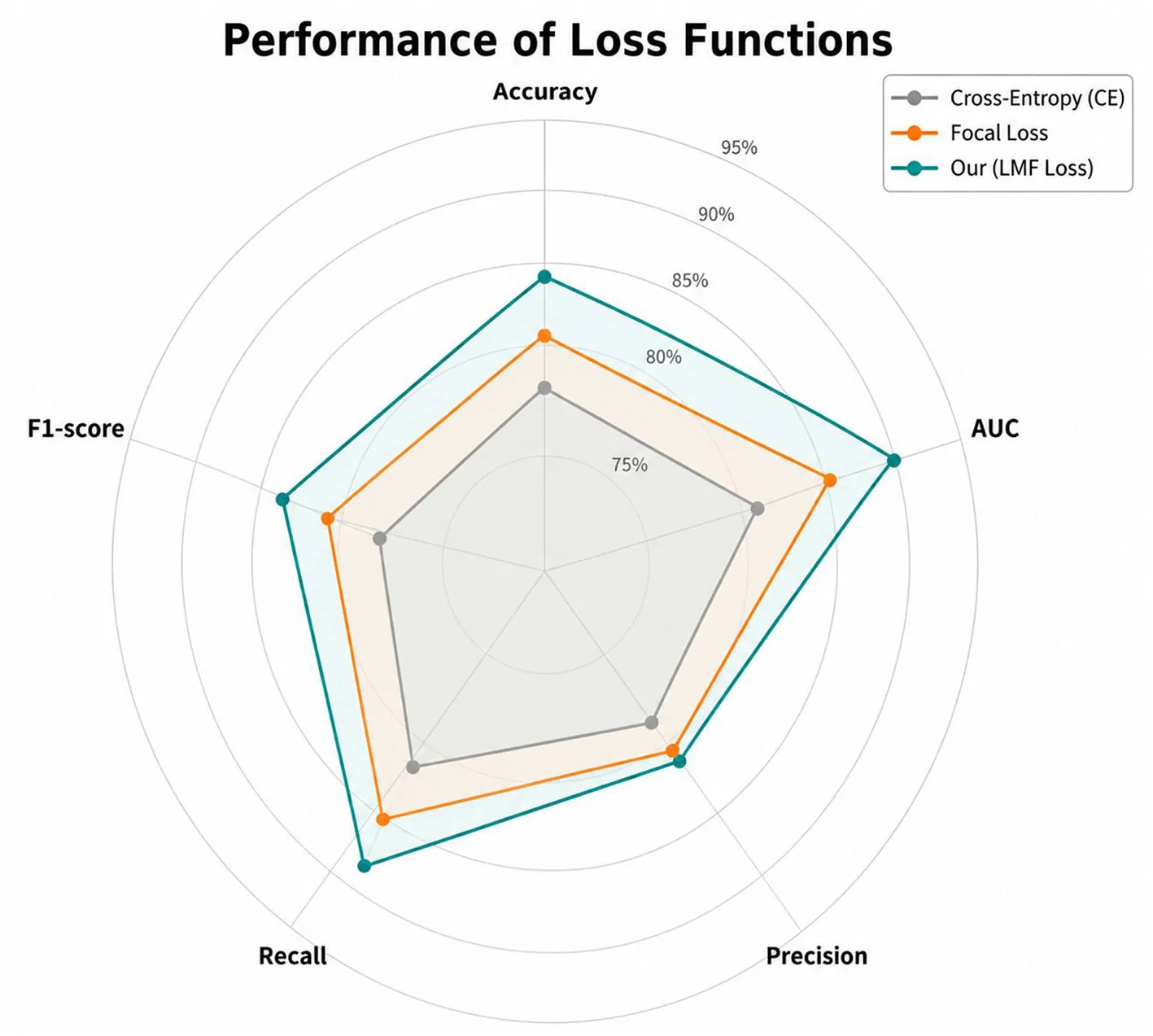
Multi-dimensional performance comparison via radar chart.

Multi-dimensional evaluation is essential to verify the overall robustness of the final model. As illustrated in Figure 5, we employed a radar chart to synthesize the performance of the three loss functions across five key diagnostic dimensions: Accuracy, AUC, Precision, Recall, and F1-score. The LMF loss enables a balanced and simultaneous escalation across all benchmarks. Notably, the expansion is most significant along the Recall and AUC axes, which directly aligns with our clinical objective of establishing a high-sensitivity screening. This holistic superiority reinforces that the proposed multimodal framework, powered by the LMF loss, is not only theoretically sound but also practically robust for complex PCOS diagnostic tasks.

## 4 Discussion

### 4.1 Interpretability of the multimodal diagnostic framework

**Figure 6:**
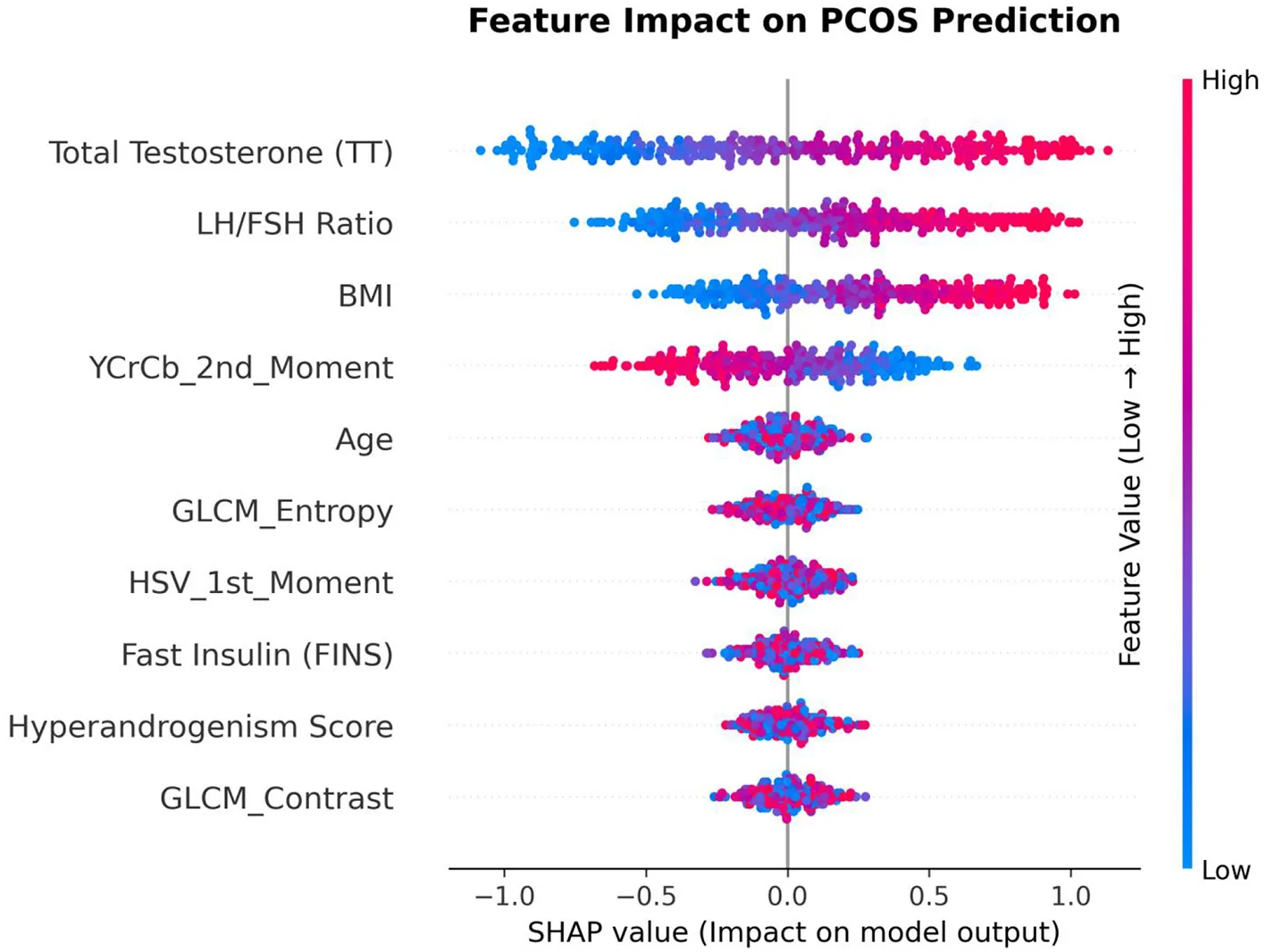
Interpretability and Clinical Logic Alignment via Multimodal SHAP Analysis.

A critical barrier to the adoption of deep learning in PCOS screening is the “black-box” nature of neural networks (19, 20). To address this, we employed SHAP (SHapley Additive exPlanations) analysis to decode the decision logic of our multimodal framework. As illustrated in Figure 6, the model's top-ranking predictors exhibit a remarkable alignment with established clinical diagnostic standards and Traditional Chinese Medicine (TCM) syndromes (25).

All variables shown in the SHAP analysis were included in the final prediction model and correspond exactly to the input variables listed in Table 2. The SHAP summary plot indicates that established clinical variables, including Total Testosterone (TT) and the LH/FSH ratio, made substantial contributions to the model output. Higher values of these variables tended to shift the prediction toward the PCOS class, which is consistent with the clinical importance of hyperandrogenism and hypothalamic-pituitary-ovarian axis dysregulation in PCOS diagnosis. In addition, BMI and fasting insulin (FINS) also showed notable contributions, suggesting that the model captured metabolic risk information that is frequently associated with PCOS. These findings indicate that the prediction process was not solely driven by image-derived patterns, but was also supported by clinically meaningful endocrine and metabolic indicators.

The tongue-image component contributed complementary phenotypic information to the multimodal model (15). In particular, color-moment features and textural entropy contributed to the model prediction, suggesting that tongue color distribution, brightness variation, and coating texture complexity may contain auxiliary information related to PCOS risk. From a clinical interpretation perspective, color-related features may reflect changes in peripheral circulation or systemic metabolic status, while GLCM-based texture descriptors may quantify coating roughness, uniformity, and complexity. These features correspond to visual characteristics that are traditionally assessed in tongue inspection, but in this study they were represented as objective digital phenotypes rather than subjective observations (17, 18).

False-positive results may lead to unnecessary anxiety and additional clinical examinations, whereas false-negative results may delay further assessment and treatment. Therefore, a positive screening result should be followed by routine gynecological evaluation, biochemical testing, and pelvic ultrasonography where appropriate. The proposed model should be regarded as an adjunctive screening tool rather than a definitive diagnostic method.

Importantly, these tongue-related features should not be interpreted as standalone diagnostic markers for PCOS (21). Instead, they serve as auxiliary external phenotypic indicators that complement established clinical evidence. The multimodal model integrates tongue-image information with clinical endocrine and metabolic variables. Therefore, although the tongue-image component is non-invasive, the complete framework should be regarded as a multimodal clinical screening and risk-stratification tool. It is intended to serve as an adjunct to routine clinical assessment rather than as a replacement for established diagnostic criteria, biochemical evaluation, or pelvic ultrasonography.

### 4.2 The role of boundary optimization in screening

The transition from Cross-Entropy loss to LMF loss represents a strategic alignment with the clinical complexities inherent in PCOS diagnosis. Standard objective functions often prioritize the optimization of high-confidence predictions, which can lead to a compressed decision boundary that fails to generalize to atypical clinical presentations. The Logit-Margin component addresses this by enforcing a stricter separation in the high-dimensional feature space. By increasing the inter-class distance between PCOS and healthy controls, the model is compelled to extract more discriminative, fine-grained representations from subtle lingual textures and hormonal fluctuations.

PCOS pathology frequently presents as a continuous clinical spectrum rather than discrete clusters, with many patients exhibiting borderline androgen levels or localized lingual microcirculatory changes. The Focal component of the LMF loss adaptively re-weights these boundary-case samples during the training phase. By modulating the loss contribution based on prediction uncertainty, the gradient updates are increasingly driven by samples residing in the proximity of the decision manifold. Consequently, the framework develops heightened sensitivity to incipient pathological signatures, such as subtle elevations in textural entropy or marginal shifts in the LH/FSH ratio, which are typically obscured in standard classification tasks.

From a diagnostic perspective, the high Recall (85.96%) facilitated by the LMF loss serves as a robust mechanism for early risk detection (21). Undiagnosed PCOS is a precursor to a cascade of metabolic and reproductive dysfunctions, including insulin resistance and ovulatory failure. By prioritizing the identification of true positive cases, the proposed framework facilitates timely intervention. This algorithmic prioritization directly supports the objectives of secondary prevention, enabling clinicians to mitigate long-term sequelae such as endometrial hyperplasia and cardiovascular complications. The efficacy of the LMF loss thus lies in its ability to transform a generalized classifier into a specialized diagnostic tool that prioritizes patient-centered outcomes over aggregate statistical accuracy.

### 4.3 Current challenges and future directions

A primary challenge resides in the inherent diversity of PCOS phenotypes across different ethnic, geographic, and clinical populations. Although the current study utilized a relatively large and high-quality single-center dataset collected under a standardized protocol, it may not fully capture the global variance in tongue manifestations, endocrine profiles, metabolic characteristics, and image-acquisition conditions. PCOS is a heterogeneous disorder, and patients may present with different phenotypic subtypes according to the Rotterdam criteria, including oligo/anovulation, hyperandrogenism, and polycystic ovarian morphology. These different phenotypes may be associated with distinct metabolic risks and external phenotypic manifestations, which could influence both tongue image features and model performance. Therefore, the current findings should be interpreted as evidence from an internal validation cohort rather than definitive proof of broad generalizability.

Future research should prioritize multi-center external validation to evaluate whether the multimodal fusion model remains robust across diverse hospitals, ethnic backgrounds, imaging devices, acquisition environments, and clinical workflows. Expanding the dataset with participants from different regions and clinical settings will be essential to reduce potential selection bias and establish a more generalizable PCOS screening model. In addition, subgroup analyses should be performed to examine model performance across different Rotterdam phenotypes, metabolic risk profiles, BMI categories, and disease severities. Such analyses would help clarify whether the learned tongue image phenotypes and clinical feature interactions remain stable across heterogeneous PCOS populations.

While the integration of tongue images and clinical biochemical markers improved diagnostic sensitivity, additional modalities could further refine the model's predictive power and clinical interpretability. Specifically, the inclusion of pelvic ultrasonography features, such as follicle count and ovarian volume, could provide a direct anatomical perspective that complements the systemic markers currently used. Additional endocrine, metabolic, and longitudinal follow-up data may also help evaluate whether the framework can support not only cross-sectional classification but also risk monitoring and early intervention. In future studies, calibration analysis, decision-curve analysis, and prospective validation should be conducted to determine whether the model provides clinically meaningful benefit in real-world screening scenarios. As this was a single-center study using an internal train-test split, the findings should be considered preliminary and may not be directly generalizable to other populations or clinical settings. External validation in larger and more diverse multicenter cohorts is required.

Finally, the practical utility of the model depends on its safe and seamless integration into clinical workflows (22). Future work will explore the deployment of the framework onto mobile and edge devices after sufficient external validation, enabling real-time point-of-care testing in community health centers and under-resourced regions. Developing a user-friendly interface that presents SHAP-based interpretability in a clinically intuitive manner will be crucial for fostering trust among healthcare providers. Importantly, the proposed framework should be regarded as an adjunctive screening and risk-stratification tool rather than a replacement for established diagnostic standards, including the Rotterdam criteria, biochemical assessment, and pelvic ultrasonography (23). This clinically cautious positioning will help ensure that AI-driven insights are effectively translated into improved patient care.

## 5 Conclusion

The tongue-image component provides phenotypic information, whereas the complete model also incorporates clinical endocrine and metabolic variables. The framework should therefore be regarded as an adjunctive multimodal screening and risk-stratification tool rather than a diagnostic system or a replacement for routine clinical assessment. This high level of sensitivity is particularly critical for clinical triage, ensuring that potential pathological signatures are identified with high fidelity.

The efficacy of the framework stems from its synergistic fusion strategy, which bridges the semantic depth of spatial-channel attention with the clinical interpretability of SHAP-based feature analysis. Notably, the system demonstrates exceptional computational efficiency, maintaining real-time processing capabilities while delivering robust diagnostic inference even in the presence of subtle or borderline pathological manifestations. This offers a transformative alternative to traditional gynecological assessments that are often constrained by subjective variability and resource intensity. Although future expansion to multi-center cohorts and integration with hospital information systems will further bolster its clinical utility, the objective and interpretable nature of this framework makes it an ideal candidate for large-scale population screening. By synchronizing advanced margin-based learning with multi-dimensional medical phenotypes, this work provides a significant advancement in the field of intelligent gynecological diagnostics.

## Data Availability

The original contributions presented in the study are included in the article/Supplementary material, further inquiries can be directed to the corresponding author.

## References

[1] Azziz R Carmina E Chen Z Dunaif A Laven JSE Legro RS . Polycystic ovary syndrome. Nat Rev Dis Primers. (2016) 2:16057. doi: 10.1038/nrdp.2016.57 27510637

[2] Ghafari A Maftoohi M Eslami Samarin M Barani S Banimohammad M Samie R. The last update on polycystic ovary syndrome (PCOS), diagnosis criteria, and novel treatment. Endocr Metab Sci. (2025) 17:100228. doi: 10.1016/j.endmts.2025.100228

[3] Fauser BCJM Tarlatzis BC Rebar RW Legro RS Balen AH Lobo R . Revised 2003 consensus on diagnostic criteria and long-term health risks related to polycystic ovary syndrome. Fertil Steril. (2004) 81:19–25. doi: 10.1016/j.fertnstert.2003.10.004 14711538

[4] Barber TM Franks S. Obesity and Polycystic Ovary Syndrome. Clin Endocrinol. (2021) 95:4–14. doi: 10.1111/cen.14421 33460482

[5] Teede HJ Misso ML Costello MF Dokras A Laven J Moran L . Recommendations from the international evidence-based guideline for the assessment and management of polycystic ovary syndrome. Clin Endocrinol. (2018) 89:251–68. doi: 10.1111/cen.13795 30024653 PMC9052397

[6] Lundberg SM Lee SI. A unified approach to interpreting model predictions. In: Advances in Neural Information Processing Systems (NIPS). (2017). p. 4765-74.

[7] Shafique M Sajid M Wang Y Ahmed B Khan S Liu T. FocusSDF: boundary-aware learning for medical image segmentation via signed distance supervision. arXiv. (2025). doi: 10.1109/ISBI61048.2026.11515937

[8] Deng H Li Y Liu X Wang Z Chen Y Zhang W . Multi-scale dual attention embedded U-shaped network for accurate segmentation of coronary vessels in digital subtraction angiography. Med Phys. (2025) 52:3135–3150. doi: 10.1002/mp.17618 39899182 PMC12082757

[9] Barrera FJ Brown EDL Rojo A Obeso J Plata H Lincango EP . Application of machine learning and artificial intelligence in the diagnosis and classification of polycystic ovarian syndrome: a systematic review. Front Endocrinol. (2023) 14:1106625. doi: 10.3389/fendo.2023.1106625 37790605 PMC10542899

[10] Bharati S Podder P Mondal MRH. Diagnosis of polycystic ovary syndrome using machine learning algorithms[C]//2020 IEEE region 10 symposium (TENSYMP). IEEE (2020) p. 1486–1489. doi: 10.1109/TENSYMP50017.2020.9230932

[11] Agirsoy M Oehlschlaeger MA. Oehlschlaeger. A machine learning approach for non-invasive PCOS diagnosis from ultrasound and clinical features. Sci Rep. (2025) 15:33638. doi: 10.1038/s41598-025-10453-9 41022847 PMC12479987

[12] He K Zhang X Ren S Sun J. Deep residual learning for image recognition. In: Proceedings of the IEEE Conference on Computer Vision and Pattern Recognition (CVPR). (2016). p. 770-8. doi: 10.1109/CVPR.2016.90

[13] Woo S Park J Lee JY Kweon IS. Cbam: Convolutional block attention module. Proceedings of the European conference on computer vision (ECCV). (2018). doi: 10.1007/978-3-030-01234-2_1

[14] Vaswani A Shazeer N Parmar N Uszkoreit J Jones L Gomez AN . Attention is all you need. In: Advances in Neural Information Processing Systems (NIPS). (2017). p. 5998-6008.

[15] Chen T Chen Y Zhou Z Zhu Y He L Zhang J. Deep learning-based automated tongue analysis system for assisted Chinese medicine diagnosis. Front Physiol. (2025) 16:1559389. doi: 10.3389/fphys.2025.1559389 40356770 PMC12066954

[16] Wu D Chen L Ma Z Zhou D Fu L Liu M . Source analysis and health risk assessment of polycyclic aromatic hydrocarbon (PAHs) in total suspended particulate matter (TSP) from Bengbu, China. Sci Rep. (2024) 14:5080. doi: 10.1038/s41598-024-55695-1 38429521 PMC10907572

[17] Diamanti-Kandarakis E Dunaif A. Insulin resistance and the Polycystic Ovary Syndrome revisited: an update on mechanisms and implications. Endocr Rev. (2012) 33:981–1030. doi: 10.1210/er.2011-1034 23065822 PMC5393155

[18] Li X Yang T Hu Y Xu M Zhang W Li F Automatic tongue image matting for remote medical diagnosis 2017 2017 IEEE International Conference on Bioinformatics and Biomedicine (BIBM) Kansas City MO USA. (2017) p. 561–564. doi: 10.1109/BIBM.2017.8217710

[19] Kranthi S Sandeep Y Pranathi K Laxmi Lydia E Joshi GP Cho W. A multimodal feature fusion with deep representation learning approach for polycystic ovary syndrome diagnosis using ultrasound images. Sci Rep. (2026) 16:9977. doi: 10.1038/s41598-026-40718-w 41721030 PMC13022374

[20] Alamoudi A Khan IU Aslam N Alqahtani N Alsaif HS Al Dandan O . A deep learning fusion approach to diagnosis the polycystic ovary syndrome (PCOS). Appl Comput Intell Soft Comput. (2023) 2023:9686697. doi: 10.1155/2023/9686697

[21] Zheng W Wu Y Chu J Jin C Qu Z Jean-Marie N . Characteristics of tongue images and tongue coating bacteria in patients with colorectal cancer. BMC Microbiol. (2025) 25:285. doi: 10.1186/s12866-025-04014-3 40350439 PMC12066039

[22] Hosseini SM Ebrahimi A Mosavi MR Shahhoseini HSh. A novel hybrid CNN-CBAM-GRU method for intrusion detection in modern networks. Results Eng. (2025) 28:107103. doi: 10.1016/j.rineng.2025.107103

[23] Chen W Miao J Chen J Chen J. Development of machine learning models for diagnostic biomarker identification and immune cell infiltration analysis in PCOS. J Ovarian Res. (2025) 18:1. doi: 10.1186/s13048-024-01583-1 39754246 PMC11697806

[24] Liu F Su D Shi X Xu S-M Dong Y-K . Cross-population tongue image features and tongue coating microbiome changes in the evolution of colorectal cancer. Front Microbiol. (2025) 16:1442732. doi: 10.3389/fmicb.2025.1442732 40012785 PMC11863330

[25] Liu Y Fan L Zhao M Wei D Zhao M Dong Y . Study on a traditional Chinese medicine constitution recognition model using tongue image characteristics and deep learning: a prospective dual-center investigation. Chin Med. (2025) 20:84. doi: 10.1186/s13020-025-01126-w 40506765 PMC12160370

[26] Lin TY Goyal P Girshick R He K Dollár P. Focal loss for dense object detection. In: Proceedings of the IEEE International Conference on Computer Vision (ICCV). (2017). p. 2980-8. doi: 10.1109/ICCV.2017.324

