# Supplementary material for: A cross-modal feature fusion model integrating tongue image phenotypes and accessible endocrine profiles for the screening of polycystic ovary syndrome
Source: Front Med (Lausanne). 2026 Aug 31;13:1858582. doi: 10.3389/fmed.2026.1858582 (PMC13572109; doi:10.3389/fmed.2026.1858582)
Supplement: Supplementary file 1 [file Data_Sheet_1.pdf]

Supplementary Material

Supplementary Table S1

Variable	Total (N=2315)	PCOS Group (n=856)	Control Group (n=1459)	*P* value
**Demographics**				
Age (years)	28.4 ± 4.2	28.2 ± 4.0	28.5 ± 4.4	0.105
BMI (Kg/m^2)	23.5 ± 3.1	24.2 ± 3.5	23.1 ± 2.8	<0.001
**Clinical Parameters**				
Menstrual Cycle (days)	–	42.1 ± 10.5	29.8 ± 2.4	<0.001
Hirsutism Score	–	5.2 ± 1.8	2.5 ± 1.1	<0.001
**Hormonal Profiles**				
Testosterone (nmol/L)	–	2.1 ± 0.8	1.6 ± 0.6	<0.001
LH/FSH Ratio	–	1.8 ± 0.7	1.2 ± 0.5	< 0.001
**Tongue Features**				
Thick Coating, n (%)	742 (32.1%)	385 (45.0%)	357 (24.5%)	< 0.001
Greasy Coating, n (%)	415 (17.9%)	210 (24.5%)	205 (14.1%)	< 0.001
Sublingual Varices, n (%)	218 (9.4%)	124 (14.5%)	94 (6.4%)	< 0.001

Supplementary Table S1. Baseline clinical characteristics and demographic distribution of the study cohort.

Note: Data are presented as mean ± standard deviation (SD) or n (%), as appropriate. Unless otherwise specified, the denominators used for the group-specific analyses were 856 participants in the PCOS group and 1,459 participants in the control group. Percentages were calculated using the corresponding group-specific denominator. A dash (–) in the Total column indicates that a pooled summary value was not presented and does not indicate missing data. All participants included in the final cohort had complete data for the variables reported in this table; therefore, no missing-value imputation was required. Continuous variables were compared using the independent-samples t-test when the data followed an approximately normal distribution; otherwise, the Mann–Whitney U test was used. Categorical variables were compared using the chi-square test or Fisher's exact test, as appropriate. All statistical tests were two-sided, and $P < 0.05$ was considered statistically significant. All statistical comparisons were re-examined using the original group-specific data and denominators. The reported descriptive statistics, frequencies, percentages, and P values were subsequently verified to ensure internal consistency.

Exclusion Criteria: Participants were excluded if they met any of the following: (i) pregnancy or lactation; (ii) other endocrine disorders (e.g., Cushing's syndrome, congenital adrenal hyperplasia, or thyroid dysfunction); (iii) lingual surface interference, such as active oral infections or recent consumption of staining foods; (iv) severe systemic diseases including NYHA Class III–IV heart failure, decompensated cirrhosis (Child-Pugh Class C), or CKD Stage 4–5; and (v) inability to cooperate with the standardized tongue image acquisition protocol.

2 **Supplementary Figure S1**

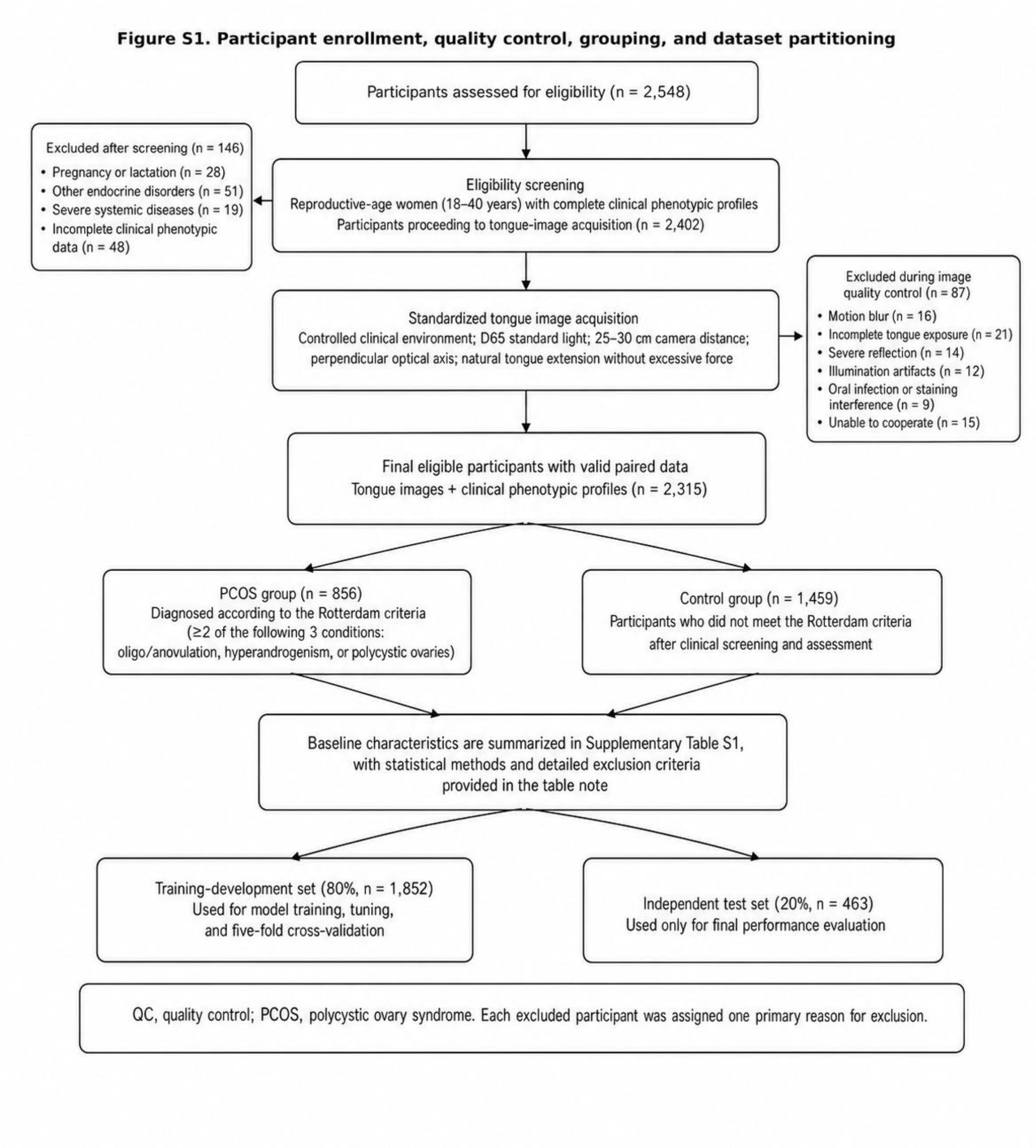

Supplementary Figure S1. Participant enrollment, quality control, grouping, and dataset partitioning.

Note: It summarizes the participant enrollment, exclusion, grouping, tongue image quality-control,

and dataset partitioning process. PCOS was diagnosed according to the Rotterdam criteria. The final dataset included 2,315 participants, including 856 PCOS patients and 1,459 controls, and was divided into a training-development set and an independent test set at a ratio of 8:2.
